# Understanding the Dementia Care Triad: Findings From a Longitudinal Qualitative Study With People Living With Dementia, Their Carers, and Their Healthcare Professionals

**DOI:** 10.1177/14713012251372224

**Published:** 2025-08-30

**Authors:** Remco Tuijt, Jill Manthorpe, Greta Rait, Rachael Frost, Jane Wilcock, Kate Walters

**Affiliations:** 1 Research Department of Primary Care and Population Health, 4919 University College London, London, UK; 2 4616 Social Care Workforce Research Unit, King’s College London, London, UK

**Keywords:** dementia, qualitative, healthcare, care network, longitudinal, relationships

## Abstract

**Background:** Best healthcare practice for people with dementia encourages the inclusion of family members or carers, alongside enabling people with dementia to make their own decisions. Dementia care thus often includes the person with dementia, their carer, and their healthcare professional (HCP): a dementia care triad. Understanding how this triad is developed and navigated may improve future dementia care services. **Methods:** A longitudinal qualitative approach was used to interview 30 people with dementia, 31 family carers, and 7 healthcare professionals in England between May 2020 and March 2021. Data from three interview time points were transcribed and analysed using reflexive thematic analysis. **Findings:** The relationships within a dementia care triad were initially built on the dyadic relationship between the person with dementia and their family carer and any pre-existing relationships with their HCP. The nature and proximity of the carer to the person with dementia influenced how triadic relationships in dementia care formed and functioned, with spousal and co-resident carers more actively involved in healthcare interactions. Further positive development of a triad required confidence in the HCP, and shared perspectives on balancing the involvement of the carer and the independence of the person with dementia, with considerations of autonomy and risk, and which did not always follow a linear transition. While increased carer involvement often supported the person with dementia, it sometimes led to their exclusion. Engagement by healthcare professionals varied, reflecting inconsistencies in applying person-centred care. **Conclusions:** The findings of this study suggest a need for clearer, more consistent approaches that support dynamic carer roles while preserving the autonomy of the person with dementia. This study provides additional considerations in relationship dynamics that inform our understanding of the dementia care triad.

## Introduction

Dementia is a neurodegenerative disorder resulting from a number of different illnesses that affects over 57 million people worldwide (Nichols et al., 2022). Post-diagnostic dementia care encourages the inclusion of a family member or care partner to supplement care planning and provision (NICE, 2018), eventually often leading to proxy decision making (Livingston et al., 2010). In this paper, any individual providing care as a family member or friend to a person with dementia, that does not receive payment, is termed a ‘carer’, as defined in legislation within England (Care Act, 2014). There is an increasing need to better understand how people with dementia and their carers navigate and engage with post-diagnostic dementia care (Wollney et al., 2024).

Best practice dementia care recommendations within the literature include person-centred care (Brooker, 2003), which encourages a focus on the individual who should be involved in their care and approached in a manner that is considerate and aware of their personal situation, their environment and their concerns, no matter the stage of their dementia (Edvardsson et al., 2008; Fazio et al., 2018). Similar approaches include relationship-centred care, where interactions between individuals serve as the foundation of care (Adams & Gardiner, 2005; Nolan et al., 2004), and family-centred care, which further encourages the inclusion of family members in the healthcare of a person with dementia (Hao & Ruggiano, 2020; Ramachandran et al., 2023). Recent frameworks combine the ideas of person-centred, relationship-centred and family-centred care encouraging and supporting the autonomy and independence of the person with dementia whilst involving and engaging with their carers (Bamford et al., 2021; Lord et al., 2020). The person with dementia, their carer, and any involved healthcare professional (HCP) may all contribute to the establishment and efficacy of support for the person with dementia, and can be referred to as members of a dementia care triad (Fortinsky, 2001).

About two-fifths of general healthcare consultations with older adults include a ‘companion’ (often a family member, but not exclusively) (Wolff & Roter, 2011), and these consultations become more carer-directed instead of patient-directed as the cognition of the patient deteriorates (Riffin et al., 2021). Carers also take on various other roles, ranging from logistical (transport, physical help) to informational (memory aid, ensuring understanding) or emotional support (Laidsaar-Powell et al., 2013). People with long-term illnesses are more likely to be accompanied for emotional and informational support, whereas for geriatric or primary care consultations, this is often logistical or informational (Laidsaar-Powell et al., 2013). Within current dementia care contexts, where cognition deteriorates, the applicability of these findings needs to be established.

Previous dyadic research confirms that when people with dementia and their carers have shared care goals, this promotes positive interactions, while if goals for the dyad are solely identified by one of them, this can increase stress for the dyad (Bosco, Schneider, Coleston-Shields, Sousa, & Orrell, 2019). Outside of healthcare interactions, carers also contribute to the agency of people with dementia (Bosco et al., 2019). Research including all members of the dementia care triad has reported on factors that influence triadic interactions, such as shared decision making (Groen-van de Ven et al., 2017; Mattos et al., 2023), but has not often included a focused triadic perspective enabling an understanding of the interplay of triadic relationships (Tuijt et al., 2021).

This paper aims to explore the experiences of people with dementia, their carers and their HCPs as they developed and navigated a dementia care triad. While it was hypothesised that triad dynamics would change as dementia progresses, as well as when living situations change (for example, moving to residential care), as a first instance this study focused on people living with mild to moderate dementia, who were living in the community.

## Methods

### Context

This study used a longitudinal qualitative interview design to explore the experiences of people with dementia, their carers and their HCPs in the community, over time during their post-diagnostic care. We have reported this here in line with high quality guidelines for reporting of qualitative studies, written up in the supplementary material (O’Brien et al., 2014).

Eligible people with dementia were recruited initially and asked to identify other triad members who were then approached. Recruitment used purposive sampling, aiming for a range of participants with varied type of dementia, gender, age, ethnicity, and living in English urban/rural locations. Participants were recruited from memory services in East London as well as primary care practices in North London and Thames Valley; supplemented by online recruitment through Join Dementia Research (National Institute for Health Research) and the Alzheimer’s Society Research Network (Alzheimer’s Society, 2020). The study received ethical approval from London South East Ethics Committee (20/LO/0006). Two Patient and Public Involvement (PPI) members were involved in the study development, design and analysis as described below.

### Recruitment Procedures

Participants with dementia were screened for eligibility using the following criteria:

(1) Diagnosis of dementia and living in the community
(2) Capacity to take part in an interview
(3) Able to complete an interview in English.

Eligible individuals were sent an invitation letter and a study information sheet, after which they could contact the study team to express interest. Initial interviews were offered individually, but if the person with dementia and their carer requested to be interviewed together, a dyadic interview was conducted. Those interviewed together were each offered the opportunity to talk individually following the interview to provide further information or follow up. HCPs were informed that one of their patients was taking part in the study and had suggested the research team invite their participation. Interviews were offered at baseline, 3 months and 6 months. If one member of the triad was not able to be recruited or retained, the other two members of the triad were retained.

### Data Collection

Interviews were conducted between May 2020 and March 2021. Due to the COVID-19 pandemic and related restrictions, data collection and consent procedures were undertaken remotely. Informed consent procedures were audio-recorded with permission with careful explanations provided to participants, point by point. Interviews were semi-structured and followed a topic guide that had been developed by the authors who were experienced qualitative researchers, clinicians and former carers (see Supplemental Material 1). The topic guide was then discussed with two PPI members, a person with dementia and a former carer, in its initial and final versions. The interviewer for all participants was a cisgender man (RT), a researcher with experience of conducting qualitative research with people with dementia, using the remote method preferred by the participants (this was via telephone for all but one dyadic interview conducted via video call). Demographic information was collected at the end of the interview, and included age, ethnicity, type of dementia, living arrangements of the dyad and relationship to each other.

### Data Processing

All interviews were audio-recorded and sent for transcription by a third-party company. Six randomly selected audio files were transcribed by the first author to aid data immersion. The first author checked all transcripts against the original audio file for any discrepancies while also pseudonymising the final transcripts. References to other participants were maintained using their relevant participant code. Transcripts were then imported into NVivo software (version 12.6) (QSR International Pty Ltd, 2020).

### Data Analysis

Reflexive thematic analysis was used to analyse the data, coding line by line and then grouping themes together where codes described similar experiences (Braun & Clarke, 2006; Braun et al., 2023). This provided an inductive approach to generating themes, which were refined and synthesised as appropriate in group discussion with all authors initially following the baseline interviews. Analysis of the further longitudinal interviews was continued by the first author, which developed new themes or expanded previously established ones. At this stage, certain themes were established between members of the triad then others were developed longitudinally (Thomson & Holland, 2003), noting any differences throughout the three time points as well as with regards to other triad members (Corden & Millar, 2007).

### Qualitative Approach and Research Paradigm

This paper presents the findings of a qualitative study within a social constructivist approach (Garrison, 1998). In this manner, the overall interpretation and meaning of each of the themes were developed through several team meetings, so each author could contribute insight from their area of expertise, including dementia, healthcare provision, caring, ageing, and qualitative research. Two PPI members were involved in the team discussion as well in individual meetings, providing contributions from their lived experience to assist the development of the themes. The team worked, for example, with the phrase “*they [person with dementia] may not understand what you are trying to explain to them*” which would initially be coded with “HCP and person with dementia”, “impact of dementia symptoms”, and “informational support” amongst others. Through further meetings this was synthesised iteratively into “symptom and risk management” alongside other codes, eventually becoming the theme presented in this paper: “*Maintaining independence while managing risk*”.

## Findings

### Participants

Interviews were conducted with 30 people with dementia and 31 carers, and seven interviews with HCPs from nine triads. Most participants identified their general practitioner (GP) as their main HCP regarding their dementia care, and four GPs were recruited. This completed six triads in total. Two NHS memory service nurses and one psychiatrist specialising in older adults were interviewed as the identified HCP in three triads. The sample of people with dementia was heterogeneous, spanning varied ages (range 68–100 years), carer relationships (spouse, adult child or friend), ethnic groups and dementia diagnoses (see Table 1).

**Table 1.** Demographics of People With Dementia (*n* = 30) and Their Carers (*n* = 31)

People with dementia		Carers	
Gender		Gender	
Female	17	Female	20
Male	13	Male	11
Ethnicity using UK census categories		Ethnicity using UK census categories	
White British	19	White British	18
South Asian	4	South Asian	5
Black Caribbean	4	Black Caribbean	4
Any other white background	3	Any other white background	4
Age (years)		Age (years)	
Range	68–100	Range	45–84
Mean	82	Mean (Spouses)	76
		Mean (Children)	57
Dementia diagnosis		Relationship	
Alzheimer’s disease	9	Child	15
Unspecified	9	Spouse	14
Mixed	7	Friend	2
Vascular	5		
Date of diagnosis (year)			
Median	2019		
Range	2011–2019		
Living status			
With carer	20		
With other family	4		
In sheltered accommodation^a^	3		
Alone	3		

^a^No care staff on site but sometimes a housing manager is present or contactable, accommodation often includes adaptations for disability.

### Retention and Attrition

At the second timepoint, 27 people with dementia and 25 carers participated (*n* = 52), and at the third timepoint, 23 people with dementia and 24 carers were interviewed (*n* = 47). Reasons for not continuing participation included deterioration in dementia related symptoms or no new information to discuss, in addition to no response to the invitation to participate further. Overall, 26/30 people with dementia and 29/31 carers were interviewed at two time points. Due to time constraints and additional pressures arising from the COVID-19 pandemic it was not possible to interview HCPs more than once.

## Themes

The characteristics of the dyads and triads in this study were found to influence experiences and relationships, and we first describe these configurations and influences. We then report on three themes relating to the dementia care triad that we developed. The first theme describes the relationship with the healthcare professional (*Which health care professional?*), and the second theme presents how triad members thought about balancing the independence of the person with dementia and potential risks related to the symptoms of dementia (*Maintaining independence while managing risk*). Finally, the third theme explores the development and progression of carers’ involvement (*Advocacy and establishing a relationship as a carer*).

### Dyad and Triad Characteristics

**Figure 1.**
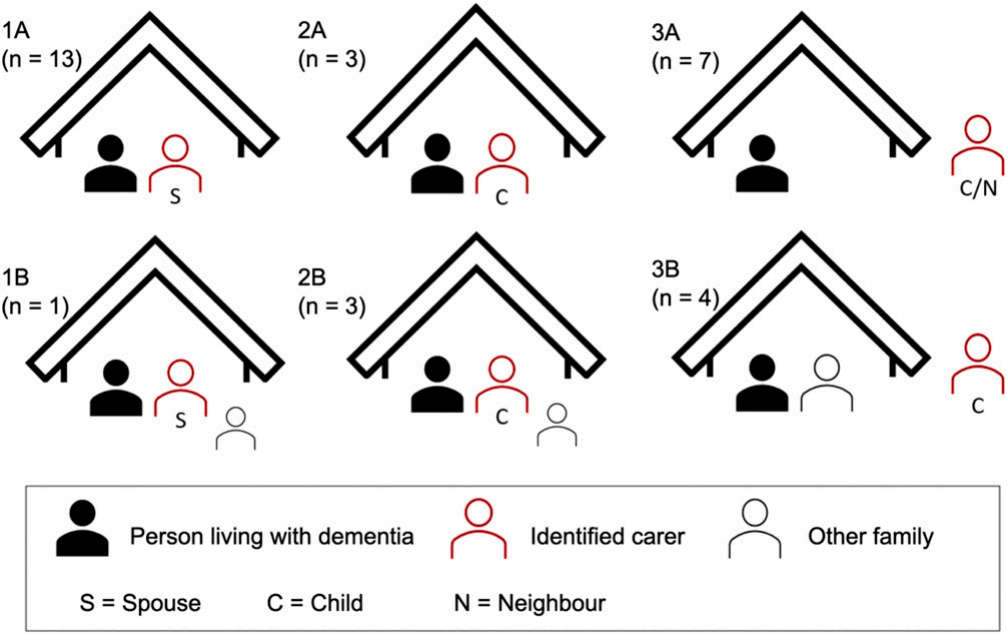
Dyad Compositions Showing Different Proximity and Relationship to Each Other

Mentions of the relationships between the members of the dyad were analysed for influential factors related to the person with dementia and their carer. From the varied characteristics of the sample, we identified two key factors that seemed to influence the dyadic relationship: proximity (living together, living with other family member(s) or not living together) and relationship type (spouse, adult child, or other). These two factors were grouped into six main dyad configurations, presented in Figure 1.

**Figure 2.**
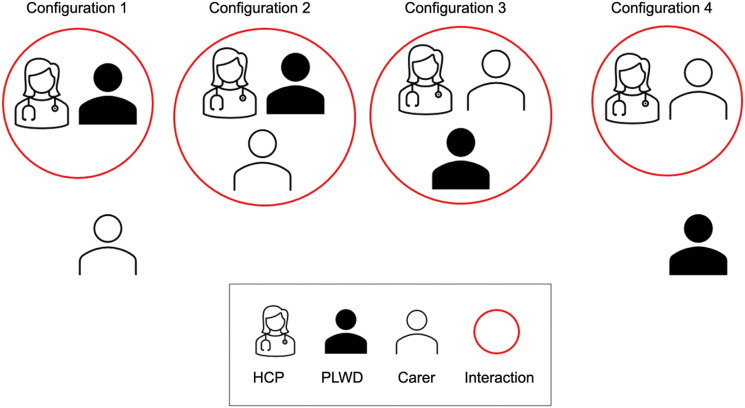
Four Examples of Triadic Interactions (HCP: Health Care Professional, PWD: Person Living With dementia, C: Carer)

An understanding of the different triadic interactions was then established, presented in a visual format in Figure 2. In some instances, the person with dementia engaged themselves with a HCP (Configuration 1) and occasionally discussed these interactions with their carer at a later point. Alternatively, the carer was present to support engagement with HCPs (Configuration 2), or sometimes led these interactions (Configuration 3). Finally, in some cases the carer would engage with the HCP on behalf of the person with dementia without them present (Configuration 4). It is important to note that lack of inclusion in one healthcare interaction (the circle in Figure 2) does not denote exclusion from future interactions.

It was common among dyads who were married and living together (1A or 1B in Figure 1) for the person with dementia to be more independent in their interactions with HCPs and in managing their own healthcare (Configuration 1). People with dementia in these situations often had a more recent diagnosis, and often presented with less severe symptoms of dementia. Practically this meant that individual or dyadic appointments (Configuration 1 or 2) with healthcare providers were considered based on health needs of both members of the dyad.

Dyads containing an adult child who lived with the person with dementia (2A or 2B in Figure 1), often had an established caring relationship over a longer period of time owing to their parents’ health problems, which often predated the dementia diagnosis. This caring relationship would start as practical support, which was often how the carer would be introduced and start engaging with the HCPs of the person with dementia. This pragmatic support would often relate to physical difficulties, such as mobility problems. In some instances, support was also being provided for dementia-related memory loss during consultations, commonly by providing information to the HCP (Configuration 2 or 3). Adult child carers often had a more defined moment in which they shifted to providing more care, more so if they were co-resident.

People with dementia who did not live with the carer they identified for this study often had established caring relationships (3A or 3B in Figure 1), where the carer would usually visit regularly to provide personal support (sometimes known as ‘distance caregiving’, in our study most carers lived within 1 hour travel of their relative with dementia). The person with dementia was often still independently engaging with their HCPs, which could be difficult for the carer to be included in, if, for example, dementia symptoms impeded efficient communication (Configuration 1). Sometimes the carer thought it necessary to speak to HCPs independently on behalf of the person with dementia (Configuration 4), often justified by their relative’s memory difficulties.

### Theme 1: Which Healthcare Professional?

People with dementia, especially if at an earlier stage of the syndrome, may not include their carer as part of their healthcare interactions. Instead, they chose to independently engage with their HCPs as they saw fit. People with dementia felt more confident speaking to HCPs with whom they had an established relationship, in this study this was often a GP they had known for some time. People with dementia who engaged with HCPs themselves usually lived on their own or were living with their spouse. Spouses were usually supportive of the person with dementia independently engaging with professionals, but noted the current symptoms of dementia were mostly mild:

I leave it to her. She’s quite capable of initiating her own call to the GP about her memory not being so good.

– Triad 31, husband, aged 80–85, individual interview at third time point

An important aspect was confidence in the relationship with the HCP, which included how comfortable an individual felt speaking to an HCP, but also how the HCP made them feel. This confidence was supported by familiarity, good communication and showing understanding:

He [GP] is amazing, and I have so much confidence in him. And he’s been extremely understanding with me, as well. [..] He’s understanding – [..] he knows me, and he’s been my doctor for quite a few years.

– Triad 28, woman with unspecified dementia, aged 70–75, individual interview at second time point

Some individuals, despite having an established relationship with their GP, highlighted secondary care professionals as more important to them due to more recent interactions or an emphasis on their specialisation and knowledge of dementia. Despite being identified as a member of a triad, the participating memory nurses highlighted the transitory nature of secondary care, where it was common to discharge a patient from their service relatively quickly. This meant that they often saw many individuals for a brief time, which they felt it was important to clarify to patients:

When we very first receive the referral from the GPs, on screening, we tend to explain what it involves in terms of the memory service, [..] the diagnosing, the treatment, and the support and everything else. They know from day one that it’s something short term, so they are already aware of this.

– Triad 9, Memory service nurse

However, they acknowledged that even after discharge, it was common for people with dementia who had more complex problems or severe presentations to return to the memory service. In our sample however there were no reports of returning to the memory service nurses, although it was noted and appreciated as an option by some carers in the relevant triads. Memory service nurses spoke of trying to clarify the system to patients:

We always say to them when they're discharged that ‘it’s not that because you're discharged you're not gonna be seen in the future. If there's any changes or any problems you go back to the GP they’ll give you a referral and we will do what we can do’.

– Triad 20, Memory service nurse

### Theme 2: Maintaining Independence While Managing Risk

Carers who indicated that they may want to start being more involved mentioned worries around how dementia symptoms might prevent optimal healthcare engagement. Some adult child carers recalled how information sharing between themselves and the person with dementia was not always optimal, but only noticed this when they spoke to HCPs:

When the doctor called me and said, ‘Oh how is your mum’s anxiety?’, I was a bit taken aback because I didn’t think it was anxiety, no-one said that, [I thought] it was to do with [a] tremor.

– Triad 5, daughter, aged 50–55, individual interview at second time point

In most of the sample, carers were regularly involved with healthcare consultations with the person with dementia. For adult children, often the first type of support they would provide for their parents with dementia was practical support in physically attending a consultation. Spousal carers had often been included in consultations prior to the onset of dementia, but for those who were not, they appreciated and recognised the importance of being included following a dementia diagnosis:

Well, the thing is, I think that someone like partners, wives, brothers, sisters who live with the person, I think they need to be included in the [dementia] advice and so. And, I was glad that [for] these things, that I was invited to join in.

– Triad 30, wife, aged 80–85, dyadic interview at first time point

If the person with dementia had concerns about their memory, they acknowledged that their carer would be able to provide information during a consultation, as well as recall discussions afterwards, which provided reassurance:

My daughter takes me to the GP. Yes, so she’s there to see me through it. She comes in with me and, because she spends a lot of time with me, so she can answer the questions almost as well as I can. [..] I feel more comfortable with her there. And, I feel that if I say something which is not quite right, she’ll put me right, because she spends a lot of time with me.

– Triad 3, woman with Alzheimer’s disease, aged 95–100, individual interview at first time point

This seemed especially helpful for people with dementia whose other members of the family were actively involved in their care, as having one carer attend meant they could share information, which is how some people with dementia were supported by multiple carers. However, as dementia symptoms progressed, some commented that having multiple carers could raise different views around decision making when trying to support the choice of the person with dementia:

C: I think because my father doesn’t feel confident anymore in these matters. So, say if he was talking to my brother, he’d be inclined to whatever my brother was suggesting. If he was talking with me, he’d be inclined to whatever I’m suggesting. […] That’s really changed hasn’t it? Because previously you’d have just made your own decisions and it wouldn’t have mattered what anybody else said.

P: Yes. But I know. I don’t know if my decision is the right decision.

C: It’s not that. Nobody knows what the right decision is now.

P: Yes but consensus.

– Triad 21, man with Alzheimer’s disease (P), aged 80-85, daughter (C), aged 40–45, dyadic interview at third time point

Carers who did not attend healthcare consultations with the person with dementia, both adult children and spouses, would often still discuss the consultations and related decisions afterwards, if the person with dementia agreed. This approach enabled the person with dementia to continue engaging with professionals as they did previously, and maintain their independence with the security of knowing they could rely on their carer if they needed:

I don’t impose on anyone if, you know, if I can do things myself [..] I’m quite independent, you know [..] what helps me to stay independent? Well knowing that I’ve got somebody to help me if I need it, that’s quite, you know, quite important to me.

– Triad 20, woman with Alzheimer’s disease, aged 80–85, individual interview at second time point

However, this appeared to be linked to the severity of dementia symptoms. Worsening symptoms could cause carers to worry whether the person with dementia was getting optimal care, encouraging their greater involvement. This was not always a clear-cut decision, as one spousal carer described feeling uncertain about whether her spouse with dementia was actively choosing not to involve her, or if this was related to her husband’s memory loss:

As I said, I don’t know if they [secondary care services] had offered him anything and he didn’t go, or didn’t remember, because as I say, he does not always share that with me.

– Triad 24, wife, aged 70–75, individual interview at first time point

If the person with dementia had more moderate or severe memory loss and lived alone, they might continue to contact HCPs about issues that had been resolved rather than include their carer:

One of the things is that she may call the surgery on her own again, for the same thing that she has already had a talk about. Not remembering that the GP has talked to her.

– Triad 1, son, aged 60–65, individual interview at second time point

For adult child carers, the provision of informational support became more prominent alongside practical support as they noticed symptoms were progressing, and would lead to a change in their role:

I think she’s lost a little bit of that overview capacity. She’s not able to look, like there’s no satellite vision of her life [..] It’s just reactive […] rather than proactive, you know?

– Triad 17, son, aged 45-50, dyadic interview at second time point

GPs reported questioning the capacity of the person with dementia depending on how severe the dementia symptoms were and if they perceived them as affecting decision making. If the GP considered there was an impact, they would engage with ‘next-of-kin’ as carers, if they were not already involved in consultations. GPs often mentioned that they would generally only make such contact with explicit consent from the person with dementia:

If my sense was that [..] they didn’t really understand, or I was concerned about whether or not they were going to be able to follow through or remember a decision that we made, I would ask their consent to discuss it with their [carers].

– Triad 29 & 30, General Practitioner

While this could be done pro-actively, only one GP mentioned specifically trying to involve the families of people with dementia early on to establish a relationship. HCPs in secondary care often seemed to have a more explicit focus on including and involving carers. This could be about enabling carers to initiate consultations, but also asking carers to be present to potentially confirm or query information being provided to the HCP by the person with dementia:

Even in the clinic, it’s the same problem [..] they [carer] have to be behind them [person with dementia], whatever they are saying they are then saying no it’s not correct. And they have to give different expressions.

– Triad 15, Psychiatrist specialising in older adults

HCPs working in secondary care related their engagement with carers to the impact of some of their patients’ symptoms that affected consultations, such as severe memory loss. One memory service nurse explained that sometimes family members explicitly request that they do not contact their relative with dementia, which could be due to more severe memory loss, or practical problems with telephone consultations:

For some patients, their relatives are quite clear at the beginning really, that the patients are not to be contacted. […] if they are further down the line in terms of their dementia, if they have, sort of, progressed a bit, it is difficult for them to kind of engage on the phone, because they may not understand what you are trying to explain to them.

– Triad 9, Memory service nurse

### Theme 3: Advocacy and Establishing a Relationship as a Carer

Some carers engaged with HCPs on behalf of the person with dementia, most commonly their adult children. Often this happened if the person with dementia had more severe symptoms that made it difficult to have consultations regarding their healthcare, including new problems or new medication. Specifically, this could also impact decision making, where the ability of the person with dementia to make decisions could be called into question. While initially this may take place as a triadic consultation, it could also lead to individual conversations between the carer and the HCP without the person with dementia. Specifically, carers who were not co-resident described it as necessary to contact HCPs individually:

And then when I did phone the doctors and get an appointment for her, my mum just said “I’m okay, thank you. I’m alright” and so I had to phone back the doctor and say “Look, she’s got dementia, she will always say she’s alright”. [...] it’s a bit going round in circles.

– Triad 15, daughter, aged 65–70, individual interview at third time point

Where carers had taken over the person with dementia’s engagement with HCPs, this was described as a more practical solution, which seemed to save time especially if the person with dementia was profoundly affected by memory loss. People with dementia who did recall the carer engaging on their behalf often described this as helpful, and even during dyadic interviews their involvement would sometimes be encouraged by the person with dementia:

INT: And [P], how do you feel about that?

P: Speak for me [C], will you, I don’t know.

Triad 10, man with mixed dementia, aged 85–90, dyadic interview at third time point (INT: interviewer; P: participant; C: carer)

There were some differences between what a person with dementia said in their interview about their regular HCP engagement and how the carer described it in their interview, mostly regarding how involved the carer had been in recent interactions or what topics had been discussed. While this discrepancy could be a difference of perspective on a recent interaction, this may be related to a symptom of dementia.

In some cases, the involvement of a carer in consultations seemed to have led specifically to exclusion of the person with dementia by the HCP. Carers who noticed this thought that this was related to ignorance around dementia, and some people with dementia described feeling belittled or patronised as a result:

When she [person with dementia] was speaking to the doctor, the doctor would look at me, or look to her son or whoever was there. And, she [doctor] would blatantly, but ignorantly, do it.

– Triad 4, female neighbour, aged 65–70, individual interview at second time point

From the perspective the HCPs, this was related to their perception (or assumption) of the severity of the dementia, as well as the capacity of the person with dementia:

If someone’s, you know, near end stage dementia, I think that’s completely different, because your priority would be talking to the family or next of kin. Whereas if someone has got mild dementia, you know, functioning day to day, I think, [..] we would always speak to- I would certainly always speak to them directly [...] I would treat them as I would any other patient.

– Triad 26, General Practitioner

The relationship between the carer and HCP of the person with dementia was also discussed, especially for those co-resident carers who were registered with the same GP. Knowing and engaging with a HCP as a patient themselves made it easier to establish a trusting relationship as a carer. If this was not the case, especially if the carer was not a relative, the carer often had to remind the GP or other staff to keep them informed. This was described as more difficult following increased remote consultations, due to the COVID-19 pandemic. Additional strain could arise if there were multiple HCPs involved, and if there was a perceived lack of communication between different HCPs:

It’s been sort of, telling people all the time at various… often the GP surgery and also the hospitals, that I’m her son, and her carer, and this is my phone number, if they have any problems they should contact me. Sometimes the link between the surgery and other hospital appointments doesn’t work very well.

– Triad 1, son, aged 60–65, individual interview at second time point

HCPs who engaged well with people with dementia were described by carers as being empathetic, able to listen to the person with dementia, while also making the carer feel included. Carers appreciated being able to speak to the HCP individually when needed, although this was not mentioned often in this sample. However, this could be at odds with the preference of the person with dementia, and might be a decision that was made by carers when considering potential risk of harm:

Actually twice I’ve come home from work and the gas is on, and that scared me and [..], to him (my father) I shouldn’t be telling everyone [at the GP] because it’s a family thing. “Family is supposed to not talk about their private business.”

– Triad 13, son, aged 60–65, individual interview at second time

## Discussion

The relationship between the dyad of the person with dementia and the carer is influenced by their relationship to each other as well as their proximity. The relationship of the carer to the person with dementia, whether spouse, adult child or friend, affected how the dyad conceptualised their caring relationship. Spousal carers were more likely to have continued involvement in healthcare interactions, sometimes joining triadic interactions prior to a dementia diagnosis, and likely had a pre-existing relationship with the HCP. Adult child carers often started with providing practical support and this relationship developed to more informational and emotional support as dementia symptoms developed. The proximity of the carer, either co-resident or not, influenced care provision, often through ease of access to healthcare consultations, as well as the structure of the caring relationship between the person with dementia and the carer as noted in other research into long-distance care (Bei et al., 2023).

When the person with dementia identified a significant HCP, it was mostly their GP, although some identified a secondary care HCP. This was related to their confidence in the existing relationship, as well as confidence in the capability of the HCP. Not all the HCPs who participated in this study reported actively engaging with carers of their patients with dementia, despite national guidance encouraging the inclusion of carers as an integral part of person-centred care (NICE, 2018). From their perspective, respecting the autonomy and independence of the person with dementia meant they did not feel the need to engage with carers unless they had concerns about decision making capacity or risks to the person with dementia. This made it difficult for some carers to become involved if they felt the person with dementia was not receiving appropriate support.

Primary care practitioners commonly had pre-existing individual relationships with the person with dementia (and potentially one with the carer). Secondary care services were more likely to take an approach that involved both the carer and the person with dementia from the start due to involving both at the point of initial assessment. Clarification of changes and developments with regard to caring roles for individuals as well as healthcare services following a dementia diagnosis has been suggested as a way to improve understanding between members of the triad (Aaltonen et al., 2021).

In our study, when carers became more involved with the healthcare of the person with dementia, they described needing to balance the autonomy of the person with dementia with the potential risks related to dementia symptoms, most commonly memory loss leading to confusion about arranging appointments, providing accurate information to HCPs or receiving treatment or advice. Differing interpretations of recent experiences may happen more often as dementia symptoms progress, which may also increase challenges for carers as they continue to adjust to and make sense of a caregiving role (Cross et al., 2018).

HCPs in our study reported similar challenges around supporting the person with dementia to be independent if they were not currently engaging with a carer as part of a triad. Most triadic relationship dynamics remained stable over the course of this study, with any recent changes notably influenced by the COVID-19 related restrictions at the time (for further COVID-19 related findings, see (Tuijt et al., 2020; Tuijt et al., 2021).

Person-centred care that highlights the personhood of the person with dementia can encourage an approach that emphasises the independence of the person with dementia, which could support continuing healthcare consultations without carer involvement (Kitwood, 1993). Some definitions of person-centred care however specifically encourage the involvement of carers (Edvardsson et al., 2008), which can create confusion regarding best practice. Critiques of person-centred care guidelines have highlighted a lack of guidance for HCPs, which emerged in our findings when HCPs spoke of attempting to find the right balance of supporting the independence and autonomy of a person with dementia whilst acknowledging the importance of the caring relationships of familial and social support (Ryan & Nolan, 2019). Developing an understanding by HCPs of the origin and development of caring relationships of their patients may help ensure support for the carer within the triad while promoting person-centred care (Fletcher, 2020).

Increased carer involvement in healthcare interactions was often described in our study as beneficial to the person with dementia, and this was often initiated as practical support by the carer such as attending appointments. As symptoms progressed, triadic consultations supported the person with dementia to remain involved in their healthcare interactions, but our participants noted that this could at times lead to their exclusion by the carer or the HCP. A previous positive relationship between the carer and the HCP seemed to make establishing a relationship as part of a triad easier, if there was continuity.

Positive relationships between the person with dementia and their HCP, similar to a therapeutic alliance (Portacolone et al., 2020), were not entirely obvious within the findings of this study. It is possible that HCPs did not have well-formed relationships with people with dementia, or that these had been disrupted by the COVID-19 pandemic. For participants who described well-developed relationships with their HCPs, confidence in the relationship between the person with dementia and the HCP was important, but this is only one of the many established influences on therapeutic alliances (Ackerman & Hilsenroth, 2003).

Practice implications arising from this study include the need for HCPs to clarify their roles in dementia care triads, whilst recognising the influence of relationship types and proximity on care dynamics. Clearer guidance, including potentially structured triadic approaches, are needed to establish a balance between supporting the autonomy of the person with dementia alongside support from carers in order to provide and strengthen collaborative, person-centred dementia care.

### Strengths and Limitations

This paper presents findings from 127 interviews undertaken with a sample of 68 participants, with up to three interviews each over a six-month period of people with dementia and their carers, with good retention across time points. This longitudinal approach enabled a richer dataset with more opportunities for people with dementia and carers to recall recent experiences with healthcare providers in their accounts over time. The time period was relatively short and influenced by the COVID-19 pandemic, so for most participants it only allowed for limited exploration of how relationships change over time with the progression of dementia, and so this was discussed by participants more generally. The sample was diverse with regards to ethnicity, age range, carer types, and gender, for people with dementia and their carers. The analysis was supported by using a team approach including individuals with extensive qualitative research experience and enhanced by engaging with PPI members with lived experience.

The limitations of the findings include a greater representation of recently diagnosed individuals, who are likely to have less frequent HCP contact due to fewer healthcare needs, as people with more moderate or severe symptoms of dementia were likely underrepresented due to potential difficulties of taking part. Additionally, all interviews were conducted in English. Our sample was limited in our recruitment of HCPs, due largely to the pressures of the COVID-19 pandemic. However, we were able to involve the relevant HCPs that the people with dementia identified in 9/30 triads. Due to the design of the study, we did not interview people with dementia who were not able to identify a carer, although no potential participants were screened out due to this. We asked the participant with dementia to identify their main carer and main HCP for interview, and did not interview multiple carers or HCPs. The relationships with HCPs may be different in these instances.

### Conclusions and Future Research

Exploration of the relationships between people with dementia, their carers, and their HCPs, in primary as well as secondary care settings, provides an understanding of some of the different dynamics between these members of the dementia care triad. This research provides valuable insights into the complexity of dementia caregiving relationships and how these impact relationships with healthcare providers.

Further research could focus on individuals who find themselves in multiple triads, including for example more than one carer or additional HCPs, or where there is no unpaid caregiver and the triad is between the person with dementia, HCP and potentially a team of paid care workers, with a remote manager. Future longitudinal research over a longer time period could provide understanding of potential trajectories within the dementia care triad and related healthcare interactions over the course of the dementia pathway, or consider opportunities to involve multiple carers or HCPs.

## Supplemental Material


Supplemental material - Understanding the Dementia Care Triad: Findings From a Longitudinal Qualitative Study With People Living With Dementia, Their Carers, and Their Healthcare Professionals
Supplemental material for Understanding the Dementia Care Triad: Findings From a Longitudinal Qualitative Study With People Living With Dementia, Their Carers, and Their Healthcare Professionals by Remco Tuijt, Jill Manthorpe, Greta Rait, Rachael Frost, Jane Wilcock and Kate Walters in Dementia

## Data Availability

The participants of this study did not give written consent for their data to be shared publicly, so due to the sensitive nature of the research supporting data are not available.
