# Supplementary material for: Understanding the Dementia Care Triad: Findings From a Longitudinal Qualitative Study With People Living With Dementia, Their Carers, and Their Healthcare Professionals
Source: Dementia (London). 2025 Aug 30;25(6):1229–47. doi: 10.1177/14713012251372224 (PMC13304905; doi:10.1177/14713012251372224)
Supplement: Supplemental material - Understanding the Dementia Care Triad: Findings From a Longitudinal Qualitative Study With People Living With Dementia, Their Carers, and Their Healthcare Professionals [file sj-pdf-1-dem-10.1177_14713012251372224.pdf]

HEARD Interview topic guide for service users

Introduction, review of consent, confirmation of permission to audio record the interview.

Present the ECOMAP, and work out a rough sketch of the current situation of the support they are receiving. Emphasise this is a draft and as you talk you can fill out or add in.

'What would you usually do on a regular day at the moment?'

'How would you say your health is right now?'

'Do you currently have any health conditions?'

'Who do you talk to about this?'

Current experiences of health care (in context of COVID-19):

Can you tell me a little bit about how you are experiencing your health care at the moment?

Have you been in touch with your health care providers? (e.g. GP/doctor, nurse, pharmacist, memory clinic)

If yes-

- In what ways are you staying connected with them?
- What was this experience (eg telephone instead of face to face) like for you?
- What did you discuss with them (e.g new concerns, ongoing issues)?
- How are you finding discussing things remotely?
- Are there any issues you are coming across?
- What went well about the experience?
- What could be improved? How?

If no-

- Why not/what is holding you back (if applicable)?
- When might you get in touch?
- Who would you contact?
- How would you get in touch if you needed to?
- What might help you access healthcare if you needed to?

SECTION 1 – INTIAL EXPERIENCE WITH POST-DIAGNOSTIC CARE

Could you tell me about your experiences of health care related to your dementia?

During the interview, prompt for:

- Health care services you have been offered and accepted or turned down
- Your original expectations and what you were looking for (was that met?)
- What you like about current services, what you dislike about services
- What you think could be improved, or if anything is missing
- Thinking ahead, do you think you might want different support from healthcare?

SECTION 2 – SPECIFIC DEMENTIA CARE

If the individual is comfortable talking about their dementia care specify as such, otherwise revert to the language they are using to describe their situation.

The following questions will be about what they felt they needed when they first received their diagnosis. Prompt to think back to their first impressions of how things were and what they are like now.

- What was your first impression of the health care services you interacted with after your diagnosis?
- Was there specific help you sought out or were directed to? What was the reason for this?
- Was there something that was offered to you that you did not end up using? What was the reason for this?
- Was there something you feel you did not get help for straight away?
- What would you recommend for someone who has recently received a diagnosis?

SECTION 3 – SPECIFIC SERVICE EXPERIENCE

If previous mention of a specific service, circle back to this and explore more.

The next experiences we are interested in are about how you feel your memory problems were addressed in [x] (your GP practice, nurses, pharmacist, health care assistants, receptionists, e.g.)

- How have you found your [x] after your diagnosis? (prompt also other professionals)
- How has your [x] been involved in any of the care you received for your memory problems? (prompt for other health problems if negative response)
- How would you like your [x] to be involved in your care?

Is there anything else you would like to tell us about the support you have received for your problems with memory and thinking?

Thank the individual, reminder about confidentiality and following interview regarding any changes next contact will be in roughly 3 months.

HEARD Interview topic guide for carers

Introduction, review of consent, confirmation of permission to audio record the interview.

May I start by asking what your relation is to the person you support? Describe the ECOMAP and update/add any additions with a different colour from the carer perspective.

‘What would you usually do on a regular day at the moment?’

‘How would you say their health is right now?’

‘Do they currently have any health conditions?’

‘Who do you talk to about this?’

What have you found difficult when providing support for [person with dementia] in the current situation?

What have you found easier when providing support for [person with dementia] in the current situation?

Could you explain anything that has changed with how you or the person you support engage with your health care providers?

- What did you discuss (new concerns vs ongoing issues)?
- How are you engaging with health care providers and how are you finding this?
- How are you finding these changes?

SECTION 1 – INTIAL EXPERIENCE WITH POST-DIAGNOSTIC CARE

Could you tell me about your experiences of health care for the dementia of the person you care for following the diagnosis?

During the interview, prompt for:

- Health care services you have initiated and have accepted or turned down
- Your original expectations and what you were looking for (was that met?)
- What you like about current services, what you dislike about services
- What you think could be improved, or if anything is missing
- Thinking ahead, do you think you might want different support from healthcare?
- Do you see the same GP most of the time? How do you find the usual appointments? Do they ask how you’re doing as well?

SECTION 2 – SPECIFIC DEMENTIA CARE

The following questions will be about what they felt was needed after the diagnosis of the person they are caring for. Prompt to think back to their first impressions of how things were and what they are like now.

- What was your first impression of the health care services you interacted with?
- Was there specific help you sought out or were directed to? What was the reason for this?
- Was there something that was offered to you that you did not end up using? What was the reason for this?
- Was there something you feel you did not get help for straight away?

- What would you recommend for someone who has recently received a diagnosis?

SECTION 3 – PRIMARY CARE DEMENTIA EXPERIENCE

If previous mention of a specific service, circle back to this and explore more.

The next experiences we are interested in are about how you feel any issues with memory problems were addressed in [x] (your GP practice, nurses, pharmacist, health care assistants, receptionists, e.g.)

- Do you accompany your relative/friend to regular appointments?
- How have you found your [x] following the diagnosis? (prompt also other professionals)
- How has [x] been involved in any of the care your relative has received for their memory problems? (prompt for other health problems if negative response)
- How would you like [x] to be involved in their care?
- How has this changed since the Covid-19 restrictions?

Is there anything else you would like to tell us about the support your relative/friend has received for problems related to their dementia?

Thank the individual, reminder about confidentiality and following interview regarding any changes first contact will be in roughly 3 months.

DEMOGRAPHICS!

AGE/ AGE OF PWD / LIVING WITH WHO? / WHAT TYPE OF DEMENTIA / ETHNICITY / HOW LONG LWD?

SNOWBALLING! ASK IF THEY CAN SHARE THE STUDY WITH SOMEONE THEY KNOW CARING FOR SOMEONE LIVING WITH DEMENTIA AT HOME. TALK TO THEM, REFER TO ME, GIVE DETAILS FOR ME TO REACH OUT, ETC.

HEARD Interview topic guide for professionals

Introductions, review of consent, confirmation of permission to audio record the interview.

Before we begin this interview, could you please confirm what your job title is?

We have explicit permission from [x], who you work with in some capacity regarding the care of their dementia. Clarify their notion of who the patient is.

COVID-19 section:

How did the ongoing healthcare for people with dementia change due to the COVID-19 outbreak?

What were the challenges? How did you overcome them?

How did a remote delivery of care work for people with dementia?

Are there any positive lessons we can take from this?

How have you found communicating and coordinating with other health care professionals regarding dementia care?

What can we learn from these recent experiences to inform how we deliver healthcare to this group now and in the future?

SECTION 1 – EXPERIENCES OF DELIVERING POST DIAGNOSTIC SUPPORT

- Tell me about what you do for [x]?
- At what point did you become involved with [x]? (how long)
- When did you last see [x]?
- What is your preferred method of communication with [x]? (carer vs pwd)
- How are you finding working with [x]?
- Are you engaging with the family of [x], and if so, how?
- Are you liaising with any other services?
- What do you think about their care? (accepting care, ease of delivering care, possible issues)
- Are there aspects you think would improve their health care?
- How did/has the way you provide care for [x] changed since Covid-19 restrictions began?

SECTION 2 – EXPECTATIONS OF POST DIAGNOSTIC SUPPORT

- What do you see for [x] going forward?
- What 3 things would you improve right now if you could?
- What is standing in the way of that?

Is there anything else you would like to say about either your service or other services for people with dementia? Is there anything that you would like to add or any questions you would like to go back to?

Thank the individual, reminder about confidentiality and potential follow up interview regarding any changes.

HEARD Topic guide follow-up interview

Introduction, review of consent and confidentiality, confirmation of permission to audio record the interview.

Establish previous contact and rapport.
'I spoke to you last [x] ago, and we discussed [x]'
Bring up main themes for their recollection as well as their approval. If there is no recollection, bring up previous themes as prompts. If there is recollection, specific language reflecting this may be used.

INTRODUCTION

- PWD: Present ECOMAP, 'I wanted to talk to you about this map we made last time I was here and how you feel about what it looks like. Has anything changed?'
- Carer: How have things been since I last saw you?
- HCP: When did you last see [x]?

REFLECTION

- 'We talked about [x] and [x], how do you feel about these things at the moment?'
- 'What could have gone better?'
- 'Was there anything regarding your care you did not expect?'

GOING FORWARD

- 'What do you think is important for you going forward?'
- 'How do you think you can manage this?'
